# Engineering Lipid Nanoparticles to Enhance Intracellular Delivery of Transforming Growth Factor-Beta siRNA (siTGF-β1) via Inhalation for Improving Pulmonary Fibrosis Post-Bleomycin Challenge

**DOI:** 10.3390/pharmaceutics17020157

**Published:** 2025-01-24

**Authors:** Xu Deng, Yingjie Yang, Liming Gan, Xinliu Duan, Xiwei Wang, Jingyan Zhang, Aiping Wang, Anan Zhang, Zhizhao Yuan, Daquan Chen, Aiping Zheng

**Affiliations:** 1 Collaborative Innovation Center of Advanced Drug Delivery System and Biotech Drugs, School of Pharmacy, Yantai University, Yantai 264005, China; dengxu881030@126.com (X.D.);; 2 State Key Laboratory of Advanced Drug Delivery and Release Systems, Shandong Luye Pharmaceutical Co., Ltd., Yantai 264003, Chinazhanganan@luye.com (A.Z.);; 3 Beijing Institute of Pharmacology and Toxicology, Beijing 100850, Chinajyzhang2023@163.com (J.Z.)

**Keywords:** lipid nanoparticles (LNPs), siRNA delivery, design of experiments (DOE), pulmonary fibrosis (PF), transforming growth factor β1 (*TGF-β1*)

## Abstract

**Background/Objectives:** Transforming Growth Factor-beta (*TGFβ1*) plays a core role in the process of pulmonary fibrosis (PF). The progression of pulmonary fibrosis can be alleviated by siRNA-based inhibiting *TGF-β1*. However, the limitations of naked siRNA lead to the failure of achieving therapeutic effect. This study aimed to design lipid nanoparticles (LNPs) that can deliver si*TGF-β1* to the lungs for therapeutic purposes. **Methods:** The cytotoxicity and transfection assay in vitro were used to screen ionizable lipids (ILs). Design of Experiments (DOE) was used to obtain novel LNPs that can enhance resistance to atomization shear forces. Meanwhile, the impact of LNPs encapsulating si*TGF-β1* (si*TGFβ1*-LNPs) on PF was investigated. **Results:** When DLin-DMA-MC3 (MC3) was used as the ILs, the lipid phase ratio was MC3:DSPC:DMG-PEG2000:cholesterol = 50:10:3:37, and N/P = 3.25; the *siTGFβ1*-LNPs could be stably delivered to the lungs via converting the *siTGFβ1*-LNPs solution into an aerosol (atomization). In vitro experiments have confirmed that si*TGFβ1*-LNPs have high safety, high encapsulation, and can promote cellular uptake and endosomal escape. In addition, si*TGFβ1*-LNPs significantly reduced inflammatory infiltration and attenuated deposition of extracellular matrix (ECM) and protected the lung tissue from the toxicity of bleomycin (BLM) without causing systemic toxicity. **Conclusions:** The si*TGFβ1*-LNPs can be effectively delivered to the lungs, resulting in the silencing of *TGF-β1* mRNA and the inhibition of the epithelial–mesenchymal transition pathway, thereby delaying the process of PF, which provides a new method for the treatment and intervention of PF.

## 1. Introduction

Pulmonary fibrosis (PF) is a life-threatening and relentless interstitial lung disease, characterized by exertional dyspnea and persistent cough. It gradually affects the interstitium, leading to impaired gas exchange, shortness of breath, reduced quality of life, and eventually respiratory failure and death [1,2]. Over the years, the incidence of pulmonary fibrosis has been on the rise, which is attributed to various factors such as occupational exposure, environmental pollution, and viral infection [3,4]. At present, the marketed drugs nintedanib and pirfenidone can only relieve clinical symptoms but cannot improve survival rates [5]. Lung transplantation is currently only effective in improving life expectancy; however, only a very small number of patients can obtain the treatment [6]. Therefore, deeper research on the pathogenesis of PF and innovative treatment are of great significance for clinical perspective.

The underlying mechanisms leading to PF have not been fully confirmed. However, it is generally suggested that it is closely related to persistent alveolar epithelial injury [7]. *TGF-β1* is the most powerful mediator in the pathogenesis of PF, which mediates the Smad pathway and plays an important role in the development of fibrosis [8]. *TGF-β1* combines with type I and type II receptors of *TGF-β* to form to activate a kinase domain, leading to phosphorylation of Smad2/3. The phosphorylated Smad2/3 forms a heterotrimeric complex with Smad4 and then undergoes nuclear translocation, which induces the expression of miRNA and inhibits the marker proteins of epithelial cells and simultaneously promotes the expression of proteins with mesenchymal cell characteristics, thereby effectively promoting alveolar epithelial–mesenchymal transition (EMT) [9,10]. Once it is translocated to the nucleus, the expression of multiple genes such as collagen, α-smooth muscle actin (*α-SMA*), and connective tissue growth factor (*CTGF*) can be modulated by the heterotrimeric complex directly binding to their promoters [11,12]. Alveolar EMT is an important sign of pulmonary fibrosis [13]. The therapeutic mechanism of both nintedanib and pirfenidone involves the *TGF-β1* [14,15]. Moreover, siRNA targeting *TGF-β1* has been proven to potentially reduce fibrosis in the kidney and heart [16,17]. Therefore, it is feasible to select *TGF-β1* as the target of siRNA to inhibit the expression of *TGF-β1* and treat pulmonary fibrosis.

However, the siRNA is a negatively charged macromolecule that neither permeated through the cell membrane nor bound to the cell surface. Moreover, challenges such as immune-stimulating effects, off-target gene silencing, and nuclease degradation need to be resolved [18]. To overcome these limitations, usually, siRNA is encapsulated in a variety of materials, including inorganic materials, proteins, lipids, polymers, and combinations of the above materials [19,20]. The first siRNA-LNP drug, Onpattro^®^ (Patisiran), was approved by the EMA and the FDA for hereditary transthyretin (TTR) amyloidosis, which targets the liver after intravenous administration [21,22]. This provides ideas for delivering si*TGF-β1* to therapy PF. However, after intravenous administration, the liver is the main organ where LNPs innately accumulate, which it cannot reach the lung to therapy [23,24]. Therefore, new administration routes or new targeting carriers need to be considered to make up for the shortage that intravenous administration cannot reach lung lesions. The complex characteristics of the lung structure provide the advantage of local rather than systemic, such as non-invasive access [25]. In terms of siRNA delivery to the lungs, there are no serum proteins in the airside; the nuclease activity remains relatively low. As a result, drugs can be delivered most effectively through inhalation and are immediately accessible to the lungs [26,27]. In addition, compared with systemic administration, local administration can reduce side effects and dosage [28]. Therefore, we consider designing LNPs that can be administered by inhalation and stably deliver si*TGFβ1* to the lungs to exert its therapeutic effect.

In this study, we present the proof of concept for a design and preclinical application of a nebulization LNP-mediated gene silencing approach targeting *TGFβ1* to inhibit the progression of PF in a bleomycin (BLM)-induced PF mouse model. First, we selected ionizable lipid (IL) types, which are closely related to the encapsulation efficiency and transfection efficiency by evaluating the encapsulation efficiency, transfection efficiency, and cell toxicity [29]. Second, we optimized the ratio of DLin-DMA-MC3(MC3), 1,2-Distearoyl-sn- glycero-3-phosphocholine (DSPC), and 1,2-dimyristoyl-rac-glycero-3-methoxypolyethylene glycol-2000 (DMG-PEG2000) in the prescription as well as the lipid phase and N/P ratio through Design of Experiments (DOE) based on the prescription of Onpattro^®^, so as to improve the stability of LNP during the atomization process [30]. Then, by comparing the content of hydroxyproline (HYP) and the content of reactive oxygen species (ROS), the expression of fibrosis-related factors such as *TGF-β1, CTGF, IL-6, IL-17, Fn, α-SAM*, and *Collagen* in BLM-induced Beas-2b cells (Human Bronchial Epithelial Cells) and the BLM-induced PF mouse model, we verified that si*TGFβ1*-LNPs can curtail the advancement of PF [31].

## 2. Materials and Methods

### 2.1. Materials

*Firefly Luciferase* siRNA (si*Fluc*), *TGFβ1* siRNA (si*TGFβ1*), and *Cy3-labeled* siRNA (si*Cy3*) were purchased from Shanghai GenePharma Co., Ltd. (Shanghai, China) DLin-MC3-DMA, DMG-PEG2000, and DSPC were purchased from Sinopeg. Cholesterol was purchased from Avanti. Quant-iT™ RiboGreen™ RNA Assay Kit was purchased from Invitrogen a company based in Carlsbad, CA, USA,. Luciferase-labeled A549 (A549-Luc) cells and human fetal lung fibroblast1 (HFL1) cells and bronchial epithelium (Beas-2b) cells were kindly provided by Luye Life Science Group. Lipofectamine RNAiMAX Reagent was purchased from Invitrogen, a company based in Carlsbad, CA, USA. Bright-GLoTM luciferase assay system was purchased from Promega. Total RNA was isolated from cells and mice lungs utilizing Trizol. Male C57BL/6 mice were procured from Jinan Pengyue Experimental Animal Breeding Co., Ltd., based in Jinan, China. TGF-β1 ELISA kits and CTGF ELISA kits were purchased from Elabscience Biotechnology, a company based in Wuhan, China.

### 2.2. siRNA-LNPs Formulation

#### 2.2.1. Preparation of siRNA-LNPs Formulations

The following lipids were prepared as stock solutions in absolute ethanol: DLin-MC3-DMA (MC3) (Sinopeg, Xiamen, China), DHA-1 (Sinopeg, Xiamen, China), SM102 (Sinopeg, Xiamen, China), ALC0315 (Sinopeg, Xiamen, China), C12–200 (Shochem, Shanghai, China), CKK–E12 (Shochem, Shanghai, China), 1,2-distearoyl-sn-glycero-3-phosphocholine (DSPC) (Sinopeg, Xiamen, China), DMG-PEG2000 (Sinopeg, Xiamen, China), and cholesterol (plant-derived) (Avanti^®^, Alabaster, AL, USA). The siRNA (either si*Fluc*, si*TGFβ1*, or si*Cy3*) stock solution was prepared in DEPC-treated water (GenePharma, Shanghai, China).

The components ILs, cholesterol, DSPC, and DMG-PEG2000 were mixed to prepare a lipid phase solution with a total lipid concentration of 15 mM. The siRNA was dissolved in 25 mM sodium citrate buffer (pH 4.0). The LNPs were prepared using an Ignite Microfluidic Rapid Nano-drug Preparation System (Micro&Nano, Shanghai, China) with a flow rate ratio of 3:1 between the siRNA solution and the lipid solution, at a total flow rate of 12 mL/min and at a temperature of 25 °C. The initial waste liquid volume was set at 0.15 mL, and the final waste liquid volume was set at 0.1 mL. The resulting siRNA-LNPs were diluted fourfold with PBS buffer solution and placed in a dialysis bag with a molecular weight cutoff (MWCO) of 300 kDa (Spectrum Medical, East Hanover, NJ, USA). The dialysis was performed for 24 h at 150 rpm in PBS buffer. Then, the siRNA-LNPs were filtered through a 0.22 μm syringe filter and transferred into vials. All the solvents used for LNP preparation were deoxyribonuclease (DNase)/RNase free.

#### 2.2.2. ILs Screening

The required amounts of various ILs ethanol solutions (MC3, SM102, ALC0315, DHA-1, C12-200, and CKK-E12), cholesterol, DSPC, and DMG-PEG2000 were measured and mixed in a molar ratio of 50:38.5:10:1.5, respectively. An appropriate volume of si*Fluc* stock solution was dissolved in 25 mM sodium citrate buffer (pH 4.0) to prepare the si*Fluc* aqueous solution, maintaining an N/P ratio of 3.25. The si*Fluc*-LNPs were then prepared following the method outlined in Section 2.2.1. The resulting si*Fluc*-LNPs were evaluated for particle size, encapsulation efficiency, cytotoxicity, and rate of luciferase activity inhibition of cells to identify the most suitable ionizable cationic lipid material [32].

#### 2.2.3. Formulation Optimization

Minitab software (https://www.minitab.com) was used to identify the parameters that may influence the mass properties of the lipid nanoparticles developed in this study, utilizing the response surface design method (Box–Behnken design). These parameters included the ratios of DLin-DMA-MC3, DSPC, and DMG-PEG2000 in the formulation, as well as the N/P ratio between the lipid phase and the siRNA aqueous phase. The experimental design involved four factors at three levels, as detailed in Table 1. The optimal formulation ratio was determined by evaluating the encapsulation efficiency and cell transfection efficiency of si*Fluc*-LNPs after atomization [33].

### 2.3. Characterization of siTGFβ1-LNPs

#### 2.3.1. Preparation of siTGFβ1-LNPs

The required ethanol solutions MC3, cholesterol, DSPC, and DMG-PEG2000 were measured and mixed in a molar ratio of 50:37:10:3, respectively. An appropriate volume of si*TGFβ*1 stock solution was dissolved in 25 mM sodium citrate buffer (pH 4.0) to prepare the si*TGFβ*1 aqueous solution, maintaining an N/P ratio of 3.25. The si*TGFβ1*-LNPs were then prepared following the method outlined in Section 2.2.1. The yielded si*TGFβ1*-LNPs were nebulized into aerosol droplets by PenWu Device for Mouse (BioJane, China) and collected in clean vial. The si*Fluc* was used to replace si*TGFβ1* to prepare si*Flu*c-LNPs for in vitro transfection assay.

#### 2.3.2. The Particle Morphology, Particle Size, and Zeta Potential of siTGFβ1-LNPs

For the transmission electron microscope (TEM), 5 μL of the samples before and after nebulization were respectively taken and dropped onto a carbon-coated copper grid. The excess liquid was blotted off with filter paper. Then, the samples were stained with 2% phosphotungstic acid, and the copper grid was air-dried. Subsequently, the samples were examined using a TEM (JEM—1400 Plus, Tokyo, Japan). The particle size and zeta potential of siTGFβ1-LNPs before and after nebulization were measured by Brookhaven Instruments (Brookhaven, New York City, NY, USA). Complexes aggregation was determined by the polydispersity index (PDI) value.

#### 2.3.3. Encapsulation Efficiency (EE%)

The encapsulation efficiency (EE%) was analyzed using the Quant-iT™ RiboGreen™ RNA Assay Kit (Invitrogen, Carlsbad, CA, USA). Briefly, using a black 96-well Costar^®^ plate (Jing’an Biological, Shanghai, China), samples were diluted to 600 ng/mL and added in a 1:1 ratio with either 1× TE buffer (for detecting free siRNA) or 2% Triton X-100 (for detecting total siRNA). RiboGreen reagent working solution was added per well as 100 μL. Fluorescence intensity was analyzed on an enzyme marker (BioTek, Winooski, VT, USA) with excitation/emission at 480/520 nm. The EE (%) was calculated using the formula: EE (%) = (Total siRNA – Free siRNA)/Total siRNA × 100%.

#### 2.3.4. In Vitro Transfection Assay

A549-Luc cells were seeded into 96-well plates with a density of 5 × 10^4^ cells per well, and 100 μL of complete culture medium was added. After 24 h, the culture medium was taken out and cells were washed twice using PBS. A total of 100 μL reduced serum medium with si*Fluc*-LNPs (0 nM, 10 nM, 25 nM, 50 nM, 100 nM, 200 nM, 400 nM, and 800 nM) was added. Four hours later, the medium was substituted with complete F-12K medium. After incubated for 48 h, the luciferase activity was detected by a microplate reader.

#### 2.3.5. Stability of TGFβ1-siRNA

The yielded si*TGFβ1*-LNPs samples were stored in a refrigerator at 4 °C, and the particle size, zeta potential, and EE% were measured on 0, 1, 3, 4, 5, and 7 days. The prepared si*TGFβ1*-LNPs samples were placed in phosphate-buffered saline with a pH of 7.4 or 6.8, and 10% fetal bovine serum (FBS) was added. Samples were taken at 0 h, 4 h, 8 h, 12 h, and 24 h, respectively, to detect the particle size [34].

#### 2.3.6. LNPs Protect siRNA from Degradation

Sodium heparin can displace the siRNA loaded by LNPs. RNase A solution can degrade siRNA. In the same conditions, (si*TGFβ1*-LNPs + sodium heparin + RNase A), (si*TGFβ1*-LNPs + RNase A), (si*TGFβ1*-LNPs + sodium heparin), and (si*TGFβ1*-LNPs) were incubated at 37 °C. After incubation, the protective effect of LNPs on siRNA was evaluated by agarose gel electrophoresis. Naked siRNA was conducted and set as the control group.

### 2.4. In Vitro Study

#### 2.4.1. Cell Culture

A549-Luc cells and HFL1 cells were propagated in F-12K Nutrient Mixture Medium (Gibco, New York City, NY, USA), and Beas-2b cells were propagated in DMEM Medium (Gibco, New York City, NY, USA), with 10% FBS and 1% penicillin-streptomycin at 37 °C and 5% CO_2_.

#### 2.4.2. Cell Viability

Cell viability was determined by the Cell Counting Kit-8 (CCK-8) (Beyotime, Shanghai, China). HFL1 cells were incubated with fresh medium containing si*TGFβ1*-LNPs at concentrations of 0, 100, 200, 400, 600, 800, 1000, 1200, and 1600 nM for 4 h, respectively. Then, cells were washed with PBS and propagated in complete medium for 24 h. And then 10 μL of CCK-8 solution was added to each well to incubate for 90 min. Using a microplate reader to measure the absorbance at 450 nm. The untreated group was used as the control to calculate cell viability.

#### 2.4.3. Cellular Uptake and Intracellular Localization

In order to evaluate the state of being taken up by cells, HFL1 cells were incubated with si*Cy3*-LNPs (400 nM). After being incubated for 2 h, 4 h, or 6 h, the intracellular location of si*Cy3* was imaged by CLSM. To explore the mechanism of cellular uptake for siRNA-LNPs, HFL1 cells were pretreated with chlorpromazine (10 μg/mL), methyl-β-cyclodextrin (M-β-CD, 57.7 μg/mL), and 5-N-ethyl-N-isopropylamiloride (EIPA, 13.3 μg/mL) for 30 min, respectively. After si*Cy3*-LNPs (400 nM) incubated for 4 h, cells were washed with PBS, and the mean fluorescence intensity (MFI) was analyzed by using flow cytometry.

#### 2.4.4. Lysosomal Escape

To evaluate endosomal escapability, HFL1 cells were incubated with si*Cy3*-LNPs for 2 h, 4 h, or 8 h. Then, the medium containing the complexes was discarded, and the cells were washed three times with PBS. Next, the cells were incubated with LysoTracker Green (BioJane, Shanghai, China) at 37 °C for 30 min to stain the lysosomes. Subsequently, the cells were fixed with 4% paraformaldehyde, and their nuclei were stained with DAPI for 15 min. After that, the intracellular location of siCy3—LNPs was imaged by CLSM.

#### 2.4.5. Wound Healing Assay

Different treatment group HFL1 cells were plated in 6-well plates with 2 × 10^5^ cells per well and then incubated overnight. Subsequently, the monolayer cells were scraped using sterile pipette tips and washed with PBS twice and then treated with si*TGFβ1*-LNPs or PBS for 4 h. Cells were washed and continued to be cultured in complete medium for 48 h, and untreated group was set as control group. The wound areas were monitored with a microplate reader at 0, 12, 24, and 48 h, respectively.

#### 2.4.6. BLM-Induced Beas-2b Cell Damage Model Established and siTGFβ1-LNPs Intervention

When the confluence of Beas-2b cells reaches 80%, Beas-2b cells were exposed to BLM (1 μg/mL) (Maokang Biotechnology, Shanghai, China) for 24 h. After BLM treatment, the cells were then treated with siTGFβ1-LNPs or PBS as the positive group for the next 24~48 h. Complete medium instead of BLM was incubated with Beas-2b cells for 24 h as a negative group (NS).

#### 2.4.7. Reactive Oxygen Species (ROS) Content Measurement

Use Reactive Oxygen Species Assay Kit (Beyotime, Shanghai, China) to detect the ROS content in different treatment group cells.

#### 2.4.8. Hydroxyproline (HYP) Content Measurement

The HYP content in different treatment group cells was determined by using a hydroxyproline assay kit (Solarbio, Beijing, China).

#### 2.4.9. Quantitative Real-Time PCR in Cells

Total RNA in different treatment group cells was isolated by the E.Z.N.A.^®^ Total RNA Kit I according to the instructions and suspended in DEPC–water. The concentration of the extracted RNA was detected using the Nanophotometer NP80 (Implen, Munich, Germany). Then, reverse transcription was performed to obtain cDNA using HiScript II Q RT SuperMix for qPCR (+gDNA wiper) (Vazyme, Nanjing, China) according to the instructions. Real-time quantitative PCR analysis was performed on the CFX96™ Optics Module (Bio-Rad, Hercules, CA, USA) using qPCR Master Mix ((Vazyme, Nanjing, China). The relative expression of specific genes was calculated based on the 2−ΔΔCT method. The details of specific primers were listed in Table 2.

#### 2.4.10. ELISA Analysis in Cells

Proteins in different treatment group cells were extracted using RIPA buffer (Solarbio, Beijing, China). Then, the release levels of TGF-β1 and CTGF in cells were detected using TGF-β1 ELISA kit (Elabscience, Wuhan, China) and Mouse CTGF (Connective Tissue Growth Factor) ELISA kit (Elabscience, Wuhan, China), respectively.

### 2.5. In Vivo Study

#### 2.5.1. Animal

C57BL/6 male mice, aged between 8 to 10 weeks, were purchased from Jinan Pengyue Experimental Animal Breeding. After the mice were anesthetized, the BLM solution dissolved in PBS was delivered to the lungs via the trachea in a single dose using a nebulizer needle device, with a dosage of 1.5 mg/kg. Meanwhile, control mice were given an equal volume of sterilized PBS. Seven days after BLM was exposed, the mice were administered via the trachea using a nebulizer needle device, receiving either sterile PBS or *siTGFβ1*-LNPs (0.45 mg/kg) once every three days. The body weight was recorded throughout experimental period. The mice were euthanized to harvest lung for subsequent experiments on day 21 after BLM exposure.

#### 2.5.2. In Vivo Tracking of siRNA-LNPs

The si*Cy3*-LNPs were nebulized into the lung of C57BL/6 mice through trachea. At 4 h, 1 day, 3 days, 5 days, and 7 days, in vitro fluorescence imaging of lungs in mice was imaged to detect the retention of si*Cy3*-LNPs in the lungs by using the Small Animal Imaging system (IVISKinetic, Waltham, MA, USA).

#### 2.5.3. Lung Coefficient

On the 21st day after administration of BLM, the mice were sacrificed. The trachea and both lungs were completely separated. The surrounding connective tissue was carefully removed. After washing with physiological saline, the lungs were blotted dry with filter paper. The wet weight of the lungs was weighed with an electronic balance, and the lung coefficient was calculated. Lung coefficient = W lung tissues /W body weight × 100%

#### 2.5.4. Histology Analysis

Lung tissue samples were processed through fixation in 4% paraformaldehyde, followed by dehydration, paraffin embedding, and sectioning for histological examination. The sections were dewaxed and stained with hematoxylin–eosin (H&E), Masson’s trichrome, and immunohistochemistry and then examined using OLYMPUS BX53M microscope (Tokyo, Japan).

#### 2.5.5. Hydroxyproline (HYP) Content

The HYP content in different treatment group mouse lungs was determined by using a hydroxyproline assay kit (Solarbio, Beijing, China).

#### 2.5.6. Quantitative Real-Time PCR in Lungs

The expression of specific genes in the lung tissues of different groups of mice was detected following the method outlined in Section 2.4.9.

#### 2.5.7. ELISA Analysis in Lungs

The expression of TGFβ1 and CTGF in the lung tissues of different groups of mice was detected following the method outlined in Section 2.4.10.

### 2.6. Biosafety Study

The si*TGFβ1*-LNPs were delivered to the lungs of C57BL/6 mice through the trachea. The nanoparticles were administered once every three days. After two weeks of continuous dosing, the mice were euthanized, and the main organs, including the heart, liver, spleen, lung, and kidney, were removed for H&E staining.

### 2.7. Statistical Analysis

GraphPad Prism 8 was utilized to conduct the data analysis, with the outcomes presented as means ± standard error. The evaluation of the statistical comparisons between two groups was conducted using the unpaired Student’s t-test (two-tailed). The levels of statistical significance were marked as * *p* < 0.05, ** p < 0.01, *** p < 0.001, **** p < 0.0001.

## 3. Results and Discussion

### 3.1. Screening of ILs for LNPs

**Figure 1:**
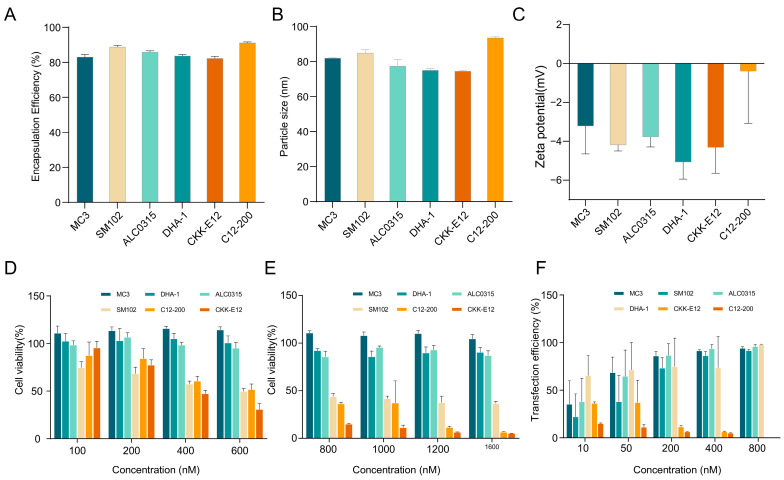
Six lipid nanoparticles (LNPs) formulations prepared with different ionizable lipids were characterized and evaluated for cytotoxicity and in vitro transfection efficiency. (**A**) The encapsulation efficiency of LNPs with different ILs (n = 3). (**B**) The particle size of LNPs with different ILs (n = 3). (**C**) The Zeta potential of LNPs with different ILs (n = 3). (**D**,**E**) The cytotoxicity of various concentrations of LNPs with different ILs in HFL1 cells from 100 to 1600 nM (n = 3). (**F**) The transfection efficiency of different concentrations of LNPs with different ILs from 10 to 800 nM (n = 3).

The lipid composition of LNP designed for siRNA delivery includes ionizable lipid, cholesterol, DSPC, and PEG–lipid with a molar ratio of 50:38.5:10:1.5. Cholesterol can enhance membrane fluidity, DSPC can promote intracellular delivery of siRNA-LNP, and PEG–lipid can prolong the circulation half-life of LNPs. The charge conversion of ILs at different pH values can achieve high encapsulation efficiency of siRNA and mediate lysosomal escape to release nucleic acids into the cytoplasm. The structure of LNPs is presented in Figure S1. However, ILs with different structures also have different toxicities to cells. Therefore, choosing the appropriate ILs is very important. In this study, six different ILs, including MC3, SM-102, ALC-0315, DHA-1, C12–200, and CKK–E12, were selected. The si*Fluc*-LNPs were prepared by the Ignite Microfluidic Rapid Nano-drug Preparation System. The particle size, zeta potential, encapsulation efficiency, cell transfection efficiency, and cytotoxicity of the corresponding LNPs were compared. As shown in Figure 1A, the encapsulation efficiency from large to small was C12-200 > SM102 > ALC0315 > DHA-1 > MC3 > CKK-E12. As shown in Figure 1B, the particle size from large to small was C12-200 > SM102 > MC3 > ALC0315 > DHA-1 > CKK-E12. The zeta potential of particles prepared by six ILs was within the acceptable range, which contributes to maintaining the stability of LNPs and enabling effective interaction with the cell membranes. (Figure 1C). The cytotoxicity from large to small was CKK-E12 > C12-200 > SM102 > ALC0315 > DHA-1 > MC3 (Figure 1D,E). And the cell transfection efficiency from large to small was C12-200 > SM102 ≈ CKK-E12 > MC3 > DHA-1 > ALC0315 (Figure 1F). Although C12-200 has the best encapsulation efficiency and transfection efficiency, it has relatively high cytotoxicity. Considering comprehensively, MC3 was selected as ILs for subsequent research.

### 3.2. Screen for Prescriptions with the Function of Being Delivered to the Lungs

**Figure 2:**
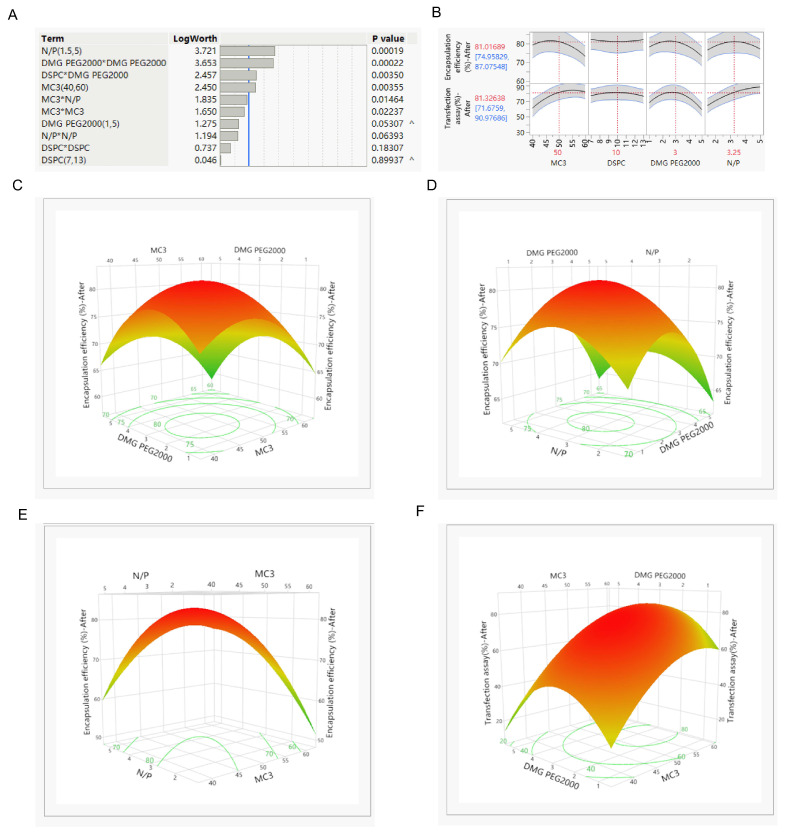

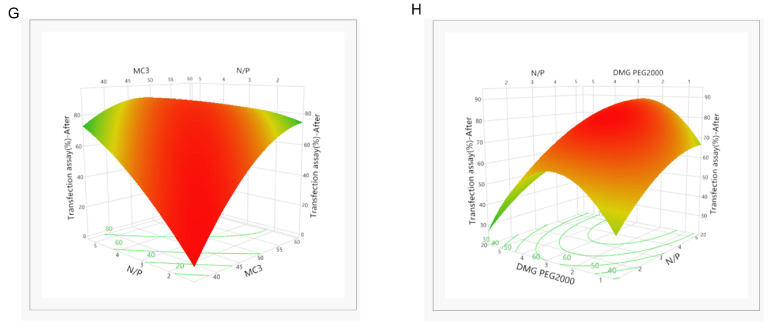
Screening of prescription Proportion of siRNA-LNPs (**A**) Pareto plot showing impact of individual and combined factors on respective responses. (**B**) Prediction profiler illustrates the optimum values for the encapsulation efficiency and transfection efficiency of LNPs after nebulization. (**C**,**F**) The 3D response surface plots illustrating the influence of the content of MC3 and the DMG PEG2000 on the encapsulation efficiency and transfection efficiency of LNPs after nebulization. (**D**,**H**) The 3D response surface plots illustrating the influence of the content of DMG PEG2000 and the ratio of N/P on the encapsulation efficiency and transfection efficiency of LNPs after nebulization. (**E**,**G**) The 3D response surface plots illustrating the influence of the content of MC3 and the ratio of N/P on the encapsulation efficiency and transfection efficiency of LNPs after nebulization.

This study aims to develop LNPs that can stably deliver siRNA to the lungs through nebulization. The stability of LNPs during nebulization and their penetrability through cellular and extracellular barriers need to be ensured. Box–Behnken was used to screen the proportions of MC3, DSPC, and DMG PEG2000 in the formulation and the N/P ratio of the lipid phase and the siRNA aqueous phase. A total of 23 formulations were prepared. The encapsulation efficiency and cell transfection efficiency after nebulization were evaluated by encapsulating *firefly luciferase* siRNA (*siFluc*) through the Ignite Microfluidic Rapid Nano-drug Preparation System. As shown in Figure 2, when the PEG–lipid ratio in the formulation is 3%, the encapsulation efficiency can reach over 80%, and it also had high transfection efficiency after nebulization. However, as the PEG–lipid prescription content increases, the encapsulation efficiency begins to decline. This indicates that increasing the PEG–lipid content helps lipid nanoparticles be more resistant to shear stress, but excessive DMG PEG2000 may reduce the encapsulation efficiency. Finally, the proportion of each component in the LNP formulation is determined as follows: MC3:DSPC:DMG PEG2000:cholesterol = 50:10:3:37, and the N/P ratio of the lipid phase and the siRNA aqueous phase was 3.25.

### 3.3. Characterization of LNPs

**Figure 3:**
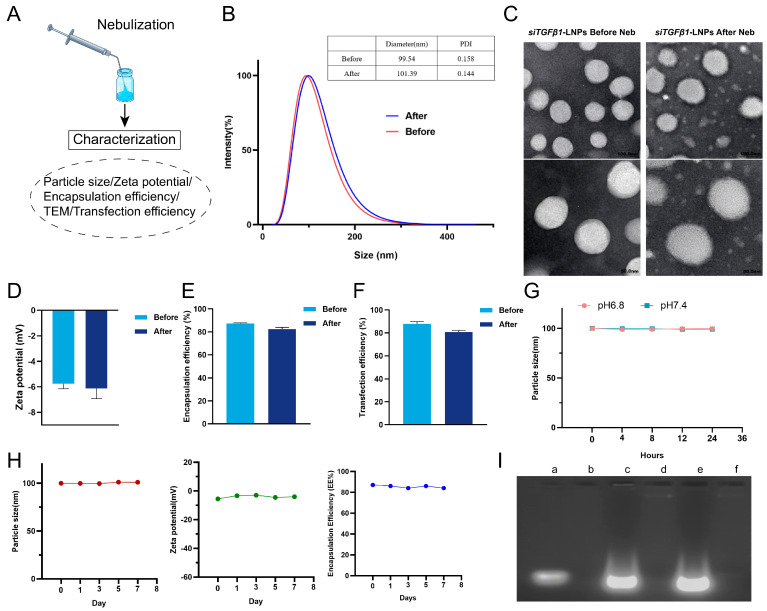
Characterization of si*TGFβ1*-LNPs post-nebulization. (**A**) Sample preparation after nebulization (**B**) Particle size before and after si*TGFβ1*-LNP nebulization (n = 3). (**C**) TEM before and after si*TGFβ1*-LNPs nebulization (Scale bar, 50 nm). (**D**) Zeta potential before and after si*TGFβ1*-LNP nebulization (n = 3). (**E**) Encapsulation efficiency before and after si*TGFβ1*-LNPs nebulization (n = 3). (**F**) In vitro transfection efficiency before and after si*Flu*-LNPs nebulization (n = 3). (**G**) Particle size of si*TGFβ1*-LNPs measured in PBS containing 10% FBS at pH6.8 or 7.4 at 37 °C for 24 h (n = 3). (**H**) Stability of si*TGFβ1*-LNPs stored in 2~8 °C (n = 3). (**I**) Gel electrophoresis assay of protective effect of lipid nanoparticles (LNPs) on siRNA (a: Naked siRNA b: Naked siRNA + RNase A c: si*TGFβ1*-LNPs + sodium heparin + RNase A d: si*TGFβ1*-LNPs + RNase A e: si*TGFβ1*-LNPs + sodium heparin f: si*TGFβ1*-LNPs).

To verify that the prepared LNPs can take the challenge of the extremely strong shear forces produced during the nebulization process. We examined whether the nanoparticle structure was damaged during nebulization. The results indicated that there were no significant changes in particle size and zeta potential of si*TGFβ1*-LNPs before and after nebulization (Figure 3B,D). Moreover, as characterized by transmission electron microscopy (TEM), the morphology of si*TGFβ1*-LNPs nanoparticles remained spherical (Figure 3C). The encapsulation efficiency after nebulization was 82.47%, which met the expected setting of more than 80% (Figure 3E). And the in vitro transfection efficiency after nebulization was 80.91, which also met the expected setting of more than 80% (Figure 3F). The si*TGFβ1*-LNPs nanoparticles’ tolerance of intense shear forces during the nebulization process renders them an ideal choice for aerosol delivery.

### 3.4. Stability of LNPs

We studied the pH impact on the stability of si*TGFβ1*-LNPs, and the results showed that there was no significant change in particle size, as the si*TGFβ1*-LNPs were incubated in PBS containing 10% FBS at pH 7.4 or 6.8, respectively, for 24 h. This indicates that LNP nanoparticles can maintain an intact structure in a mildly acidic environment in lung tissue (Figure 3G). We also studied the placement stability of si*TGFβ1*-LNPs, which showed that the yielded nanoparticles were placed at 4 °C for 7 days, and there was no significant change in encapsulation efficiency (EE%), particle size, or zeta potential (Figure 3H).

Preventing small interfering RNA (siRNA) from degradation during the delivery process is of crucial importance for ensuring its efficient gene silencing ability. As shown in Figure 3I, after incubation with a concentration of 0.2 μg/mL RNase A for 30 min, naked si*TGFβ1* showed extremely poor stability and was completely degraded. However, the si*TGFβ1*-LNP, which was incubated with a concentration of 0.2 μg/mL RNase A for 30 min, when siRNA was released by 2000 μg/mL of heparin sodium, si*TGFβ1* still exists stably. This indicated that LNP significantly improves the stability of siRNA.

### 3.5. In Vitro

#### 3.5.1. Cytotoxicity and Transfection Assay

**Figure 4:**
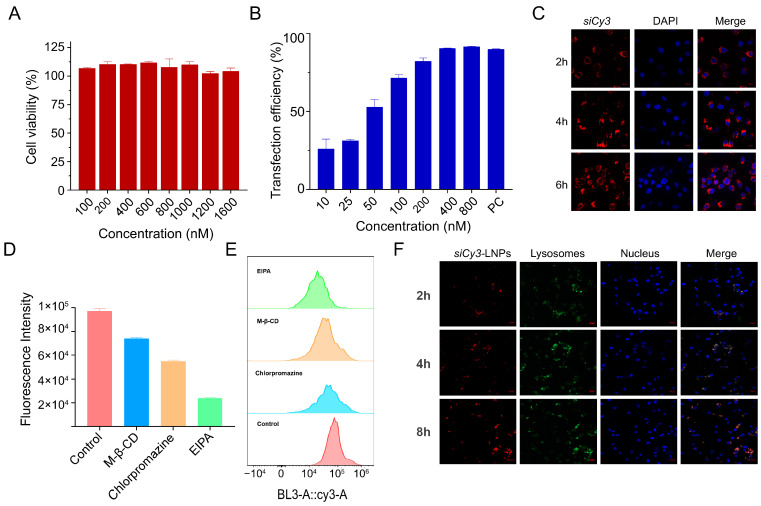
Cytotoxicity and mechanisms of si*TGFβ1*-LNPs in vitro. (**A**) The cytotoxicity of various concentrations of si*TGFβ1*-LNPs in HFL1 cells from 100 to 1600 nM (n = 3). (**B**) The transfection efficiency of different concentrations of si*TGFβ1*-LNPs from 10 to 800 nM (n = 3). (**C**) Cellular uptake of si*Cy3*-LNPs in HFL1 cells. (**D**,**E**) Effect of different endocytosis inhibitors on the cellular uptake of si*Cy3*-LNPs. (**F**) Endosomal escapability of si*Cy3*-LNPs against HFL1 cells (the fluorescence signals of blue, green, and red represent the cell nucleus, lysosome, and si*Cy3*, respectively).

In order to investigate whether the increase in the prescription ratio of DMG-PEG2000 has an impact on the toxicity of LNP, the toxicity of different concentrations of si*TGFβ1*-LNPs in HFL1 cells was evaluated. As shown in Figure 4A, the si*TGFβ1*-LNPs nanoparticles have almost no toxicity to HFL1 cells in the concentration range of 100 nM to 1600 nM.

The transfection effect of siRNA is a key process for the success of verifying gene functions or conducting gene silencing experiments at the cellular level. Therefore, choosing an appropriate siRNA concentration is crucial for the experimental results. As shown in Figure 4B, when the siRNA concentration is greater than 400 nM, the in vitro transfection efficiency exceeds 80%.

#### 3.5.2. Cellular Uptake Pathway Investigation

The nanocomplex’s cellular uptake pathway is of crucial importance for the final transfection efficiency. The cellular uptake of si*Cy3*-LNPs was assessed in HFL1 cells. When si*Cy3* was encapsulated in LNPs (si*Cy3*-LNPs), pronounced uptake was observed by confocal images after incubation with cells for 4 h (Figure 4C). In order to determine the cellular uptake mechanism of nanoparticles, flow cytometry was used to test the cellular internalization of si*Cy3*-LNPs nanoparticles in HFL1 cells pretreated with different inhibitors. As shown in Figure 4D,E, treatments with EIPA, chlorpromazine, and M-β-CD all had a significant impact on the cellular uptake of the complex. The cellular uptake rates of si*Cy3*-LNPs decreased by 74.94%, 45.73%, and 25.84%, respectively. This indicates that macropinocytosis, clathrin-mediated endocytosis, and caveolin-mediated endocytosis are all involved in the cellular uptake of si*Cy3*-LNPs nanoparticles. Among them, macropinocytosis has the greatest impact, followed by clathrin-mediated endocytosis, and finally caveolin-mediated endocytosis.

#### 3.5.3. Research on Lysosomal Escape

Usually, internalized siRNA was readily degraded by lysosomal enzymes like acid RNase and exonucleases, resulting in reducing its effectiveness. Therefore, the lysosomal escape ability of siRNA-loaded complexes is crucial for improving therapeutic efficacy. The si*Cy3*-LNPs, in which si*Cy3* was encapsulated in LNPs, were used to study the lysosomal escape ability in HFL1 cells by using CLSM. From the CLSM images (Figure 4F), there was an orange signal area, which is the overlapping area of red fluorescence (si*Cy3*-LNPs) and green fluorescence (lysosome) after incubation with HFL1 cells for 4 h, indicating that si*Cy3*-LNPs were trapped in endosomes. After incubation with HFL1 cells for 8 h, bright red fluorescence can be observed from the CLSM image. The results show that LNPs can promote endosomal escape and improve the silencing efficiency of siRNA.

#### 3.5.4. Wound Healing

**Figure 5:**
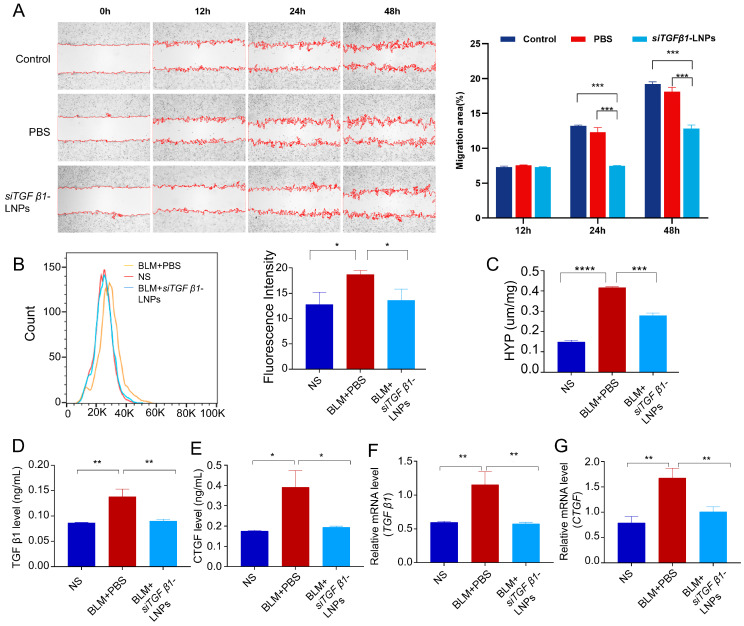
Anti-fibrosis effect of si*TGFβ1*-LNPs in vitro. (**A**) Migration of HFL1 cells at different time points after different treatment (n = 3). (**B**) Measurement of ROS of Beas-2b cells after different treatment (n = 3). (**C**) HYP content measurement of Beas-2b cells supernatant after different treatment (n = 3). (**D**,**E**) ELISA analysis of the inflammation-related factor TGFβ1 and CTGF expression of Beas-2b cells supernatant after different treatment (n = 3). (**F**,**G**) qPCR analysis of the inflammation-related factor *TGFβ1* and *CTGF* expression of Beas-2b cells supernatant after different treatment (n = 3). *, *p* < 0.05; **, *p* < 0.01; ***, *p* < 0.001; ****, *p* < 0.0001.

Considering that the development of fibrosis also involves migration of fibroblasts and myofibroblasts into fibroblastic foci. We investigated the effect of si*TGFβ1*-LNPs on fibroblast migration through the wound-healing assay. HFL1 cells were treated with PBS or si*TGFβ1*-LNPs separately for 4 h, and healing process images were taken at 0, 12, 24, and 48 h. As shown in Figure 5A, at 24 h, the wound healing percentage in the si*TGFβ1*-LNPs group was significantly lower than that in the other two groups.

#### 3.5.5. Reactive Oxygen Species (ROS) and Hydroxyproline (HYP)

The balance of ROS content maintains the normal physiological function of cells. It was reported that *TGF-β1* increases ROS production. Flow cytometry was used to measure the production of reactive oxygen species (ROS) in different treatment group cells. As shown in Figure 5B, the level of ROS in BLM-induced cells was significantly increased, while the level of ROS in the si*TGFβ1*-LNP group was significantly reduced, proving that si*TGFβ1*-LNP has a good inhibitory effect on the production of ROS in cells.

The HYP content in the samples, which serves as an indicator of fibrotic activity and collagen tissue metabolism. Measuring the total content of HYP is an important indicator for evaluating the severity of fibrosis. Therefore, the content of HYP in different group cells was detected to evaluate the severity of fibrosis. As shown in Figure 5C, compared with the control group, the content of HYP of the BLM group was significantly increased. While the content of HYP in the siTGFβ1-LNP group was significantly lower than that in the BLM group, which proves that siTGFβ1-LNP has a good inhibitory effect on the excessive synthesis of extracellular matrix proteins caused by damage from BLM.

#### 3.5.6. Inflammation-Related Factor TGFβ1 and CTGF Expression

The expression levels of TGF-β1 and CTGF in cells of different groups were detected using the TGF-β1 ELISA kit and CTGF ELISA kit, respectively. The results shown in Figure 5D,E, which indicate that the expression of TGF-β1 and CTGF was upregulated in BLM-induced cells. Compared with the control group, the concentration of TGF-β1 was increased by 1.592 times (BLM + PBS) and 1.038 times (BLM + si*TGFβ1*-LNPs), and the concentration of CTGF was increased by 2.112 times (BLM + PBS) and 1.272 times (BLM+ si*TGFβ1*-LNPs). The qPCR detection of *TGFβ1* and *CTGF* expression was consistent with ELISA results, which compared with the control group, the concentration of TGF-β1 was increased by 1.928 times (BLM + PBS) and 1.018 times (BLM+ *siTGFβ1*-LNPs), and the concentration of CTGF was increased by 2.229 times (BLM + PBS) and 1.104 times (BLM+ *siTGFβ1*-LNPs) (Figure 5F,G). This further confirms that siTGFβ1-LNPs can demonstrate a significant capacity to suppress the excessive synthesis of extracellular matrix proteins triggered by BLM-induced injury.

### 3.6. In Vivo

#### 3.6.1. Pulmonary Retention of siCy3-LNPs After Nebulization

**Figure 6:**
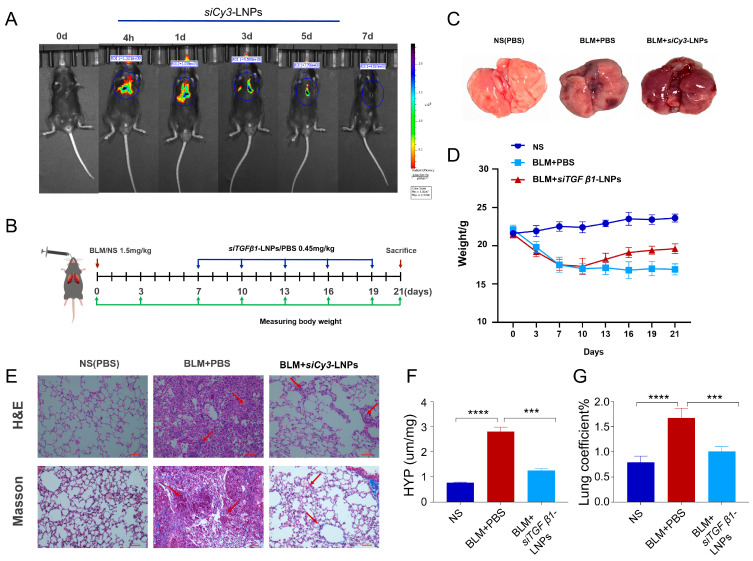
The si*TGFβ1*-LNPs mitigated BLM-induced damage to lung structure and inflammation. (**A**) In vivo imaging of the mice at 4 h and 1, 3, 5, and 7 days after si*Cy3*-LNPs nebulization. (**B**) Experimental design of mouse study. (**C**) Lung tissue morphology of mice. (**D**) Body weight changes in mice during entire experimental cycle (n = 5). (**E**) Histological analysis of lung sections, including H&E staining and Masson’s trichrome staining, scale bars, 100 μm (n = 5). (**F**) HYP content measurement of lungs in different groups (n = 5). (**G**) The lung coefficient in different groups (n = 5). ***, *p* < 0.001; ****, *p* < 0.0001.

The retention of si*Cy3*-LNPs in the lungs of C57BL/6 mice was studied within a week using the Small Animal Imaging system to ascertain the expression of siRNA in the lungs after nebulization and to determine the frequency of administration. The results showed that nebulized intratracheal administration could stably deliver nanoparticles to the lungs, and the fluorescence signal gradually weakened after day 3 (Figure 6A). Therefore, the administration frequency was selected as once every three days.

#### 3.6.2. siTGFβ1-LNPs Mitigate BLM-Induced Pulmonary Fibrosis

To test the influence of si*TGFβ1*-LNPs nanoparticles on pulmonary fibrosis, we created the mouse pulmonary fibrosis model by administering a single optimized dose of BLM intratracheally to C57BL/6 male mice. Throughout the entire study, body weight was recorded as the measure of disease burden (Figure 6B). From day 7 to day 21, mice were treated with si*TGFβ1*-LNPs nanoparticles at a dose of 0.45 mg/kg by inhalation using a nebulizer twice a week. On day 21, mice were sacrificed to evaluate the therapeutic outcomes.

On the 21st day after BLM stimulation, as shown in Figure 6C, the lungs of mice in the control group were pink, with neat and sharp edges, a smooth surface, and good elasticity. In the BLM group, the lungs were darker, with irregular edges, poor elasticity, and relatively hard texture. The lungs of the treatment group were better than those of the BLM group, which color is slightly darker than that of the normal group, and the elasticity was acceptable.

Weight monitoring revealed that compared with the control group, the body weight of mice in the BLM group decreased significantly. In the si*TGFβ1*-LNPs treatment group, the body weight of mice gradually recovered after a decrease, indicating that si*TGFβ1*-LNPs treatment can improve BLM-induced weight loss (Figure 6D). It suggests that weight loss in mice might occur after BLM induction, and si*TGFβ1*-LNPs can ameliorate this process.

The results of hematoxylin–eosin (H&E) staining showed that in mice after BLM stimulation, the alveolar walls were thickened and collapsed, and the lung tissue structure was disordered. While in mice treated with si*TGFβ1*-LNPs, the severity of fibrosis induced by BLM was greatly reduced (Figure 6E). The results of Masson staining indicated that after receiving treatment, the collagen deposition and parenchymal destruction in the lung tissues of mice were significantly reduced (Figure 6E). The results of hydroxyproline (HYP) content detection were consistent with the histological analysis. The HYP content in the lungs of mice in the BLM group was significantly increased, while after treatment with si*TGFβ1*-LNPs, the HYP content in the lungs of mice was significantly decreased, which revealed that si*TGFβ1*-LNPs can delay the process of pulmonary fibrosis in mice (Figure 6F). The lung coefficient is one of the indicators reflecting the degree of pulmonary fibrosis. After pulmonary fibrosis, the volume of lung tissue decreases, while collagen deposition makes the texture harder. As a result, the weight of the lung increases, and the lung coefficient relatively becomes larger. The results of lung coefficient in different groups of mice (Figure 6G) also indicated that collagen deposition might occur after BLM induction, and si*TGFβ1*-LNPs can slow down this process.

**Figure 7:**
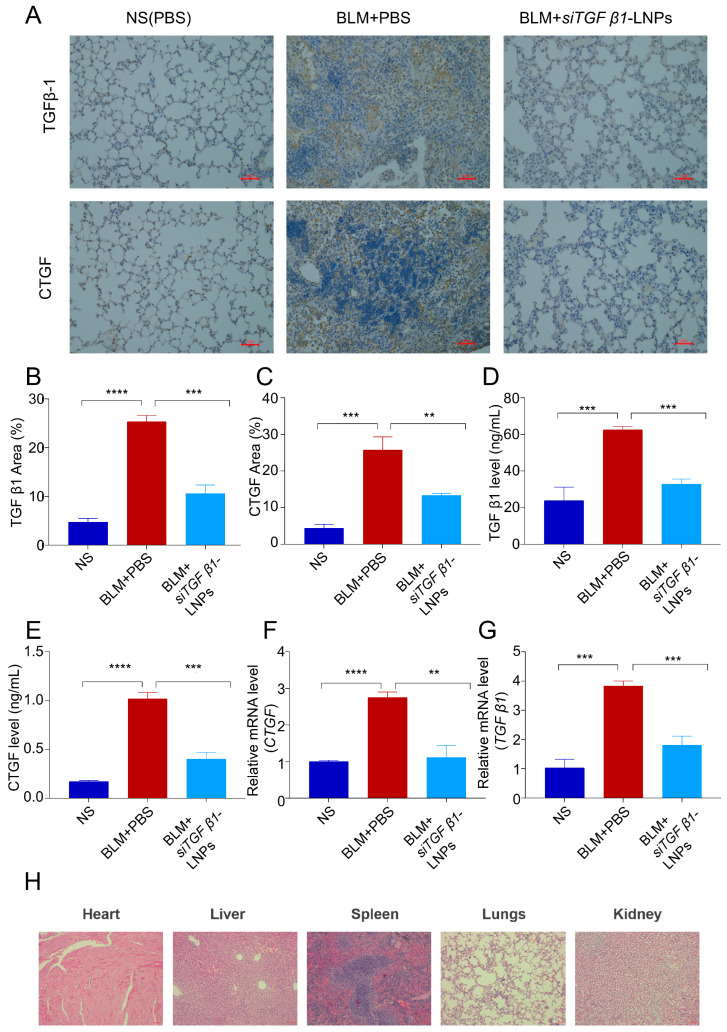
Inflammation-related factor *TGFβ1* and *CTGF* expression and toxicity evaluation. (**A**,**B**) and (**C**) Immunohistochemical staining for TGFβ1 and CTGF in different groups (n = 5), scale bar: 100 μm. (**D**,**E**) ELISA analysis of the inflammation-related factor TGFβ1 and CTGF expression in different groups (n = 5). (**F**,**G**) qPCR analysis of the inflammation-related factor *TGFβ1* and *CTGF* expression in different groups (n = 5). (**H**) Histological H&E staining of major organs (heart, liver, spleen, lung, and kidney) scale bar: 100 μm. **, *p* < 0.01; ***, *p* < 0.001; ****, *p* < 0.0001.

Immunofluorescence staining images showed that compared with the control group, BLM induction significantly increased the expression of TGFβ1 and CTGF in the lungs of mice. After treatment with si*TGFβ1*-LNPs, TGFβ1 and CTGF in the lungs of mice were significantly reduced (Figure 7A–C). Consistent with the immunofluorescence results, ELISA detection showed that compared with the control group, BLM induction upregulated the expression of TGFβ1 in the lungs of mice by 3.717 times and the expression of CTGF by 6.034 times. After treatment with siTGFβ1-LNPs, TGFβ1 in the lungs of mice was upregulated by 1.381 times and CTGF upregulated by 2.365 times (Figure 7D,E). As shown in Figure 7F,G, the qPCR detection of *TGFβ1* and *CTGF* expression was also consistent with results, which compared with the control group, BLM induction upregulated the expression of *TGFβ1* in the lungs of mice by 3.717 times and the expression of *CTGF* by 2.737 times. After treatment with si*TGFβ1*-LNPs, *TGFβ1* in the lungs of mice was upregulated by 1.761 times and *CTGF* upregulated by 1.108 times. These results indicate that si*TGFβ1*-LNPs significantly inhibited the expression of *TGFβ1* and *CTGF* in lung tissue, which can delay the process of pulmonary fibrosis in mice induced by BLM.

### 3.7. Toxicity Evaluation of Heart, Kidney, Liver, and Spleen

To further evaluate the safety of si*TGFβ1*-LNPs in vivo, histological analysis of the major organs of mice was performed. As shown in Figure 7H, no obvious toxicity-related lesions were observed in the heart, liver, spleen, lung, and kidneys of mice treated with si*TGFβ1*-LNPs. The results indicate that si*TGFβ1*-LNPs show high biosafety to the normal organs.

### 3.8. The siTGFβ1-LNPs Mitigated BLM Toxicity by Suppressing EMT Through TGFβ/Smad2/3 Signaling

To explore the potential mechanism of si*TGFβ1*-LNPs against the toxicity of BLM, we verified the knockdown efficiency of *TGF-β1* and *CTGF* mediated by siRNA. We further evaluated the expression levels of key epithelial–mesenchymal transition (EMT)-related molecules in cell cultures and animal models.

**Figure 8:**
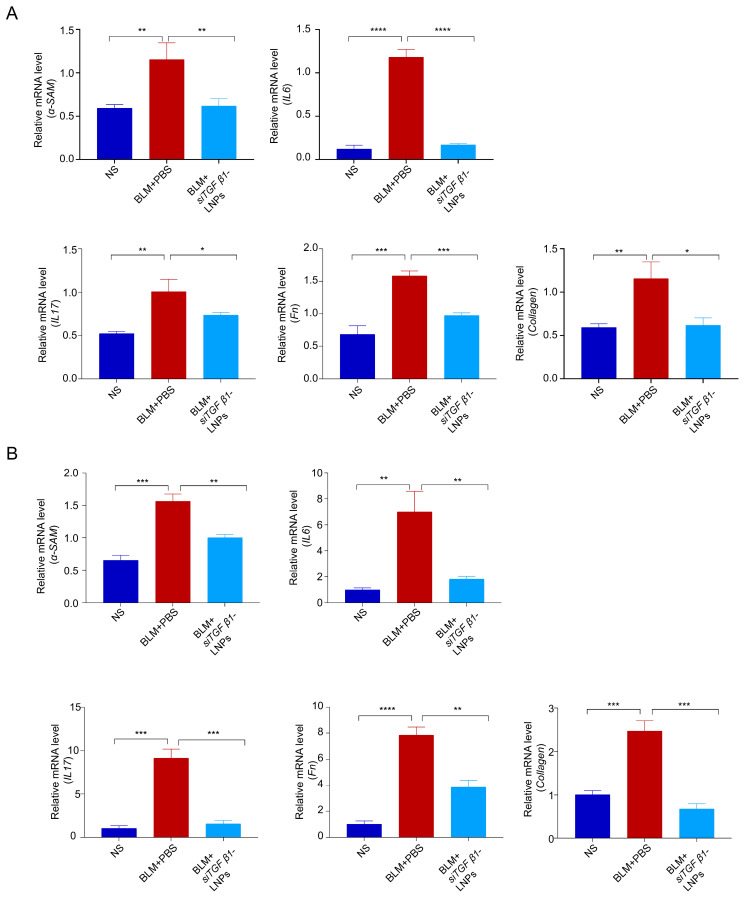
The si*TGFβ1*-LNPs inhibit the expression of factors related to *TGFβ*/Smad2/3 signaling. (**A**) qPCR detection of *IL-6*, *IL-17*, *α-SAM*, *Fn*, and *Collagen* mRNA expression in Beas-2b cells (n = 3). (**B**) qPCR detection of *IL-6*, *IL-17*, *α-SAM*, *Fn*, and *Collagen* mRNA expression in lung tissues (n = 5). *, *p* < 0.05; **, *p* < 0.01; ***, *p* < 0.001; ****, *p* < 0.0001.

BLM-induced fibrosis may lead to the upregulation of related protein factors such as *TGF-β1*, *CTGF*, *IL-6, IL-17*, *α-SAM*, *Fn,* and *Collagen* in the epithelial–mesenchymal transition (EMT) pathway. Among them, *TGF-β1* may be upregulated during the fibrosis process, promoting the activation of fibroblasts and the production of collagen, thereby exacerbating fibrosis. *CTGF* is a downstream effector molecule of *TGF-β1* and is also upregulated during the fibrosis process, thereby promoting the proliferation of fibroblasts and the production of collagen. *Interleukin-6* (*IL-6*) may also be upregulated in pulmonary fibrosis. Thus, it participates in the fibrosis process by promoting inflammatory responses and fibroblast activation. Cytokines such as *TGF-β1* and *IL-6* can promote the differentiation of naive T cells into Th17 cells, thereby inducing more Th17 cells to produce *Interleukin-17 (IL-17)*. Therefore, *IL-17* may also be upregulated. *α-SMA* is a marker of activated fibroblasts. During the fibrosis process, *α-SMA* may be upregulated, thereby promoting the transformation of fibroblasts into myofibroblasts. *TGF-β1* can strongly stimulate fibroblasts to synthesize extracellular matrix components, including *fibronectin* (*Fn*), leading to its upregulation. *Collagen* is also upregulated during the fibrosis process, leading to tissue stiffness and structural abnormalities. As shown in Figure 8A, compared with normal cells, in BLM-induced cells, the expressions of factors such as *TGF-β1*, *CTGF*, *IL-6*, *IL-17*, *α-SAM*, *Fn*, and *Collagen* are significantly increased. However, in cells treated with si*TGFβ1*-LNPs, the mRNA levels of genes such as *TGF-β1*, *CTGF*, *IL-6*, *IL-17*, *α-SAM*, *Fn*, and *Collagen* are significantly lower than those in the BLM-induced cells. Analysis of BLM-induced mice showed results consistent with cell experiments (Figure 8B). In mice treated with si*TGFβ1*-LNPs, the mRNA levels of genes such as *TGF-β1*, *CTGF, IL-6*, *IL-17*, *α-SAM*, *Fn*, and *Collagen* were significantly lower than those in BLM-induced mice. This proves that si*TGFβ1*-LNPs have an excellent inhibitory effect on the fibrosis process, which can delay the process of pulmonary fibrosis through the EMT pathway.

One limitation of this study is the absence of certain negative control groups, specifically siRNA only and LNP only, in both the in vitro and in vivo experiments. While efforts were made to comprehensively evaluate the effects of siRNA-LNPs, these controls would provide further insights, particularly into the individual contributions of siRNA and LNPs to the observed outcomes. Given the logistical and ethical challenges of incorporating additional controls into in vivo experiments, these controls should be prioritized for in vitro studies in future investigations, especially in inflammation-related studies where the potential for non-specific effects is higher.

## 4. Conclusions

In this study, we constructed the LNP delivery system capable of stably delivering siRNA to the lungs via nebulization. The LNP delivery system we designed has high safety, high si*TGF-β1* loading efficiency, high stability during the nebulization process, and high penetrability through cellular and extracellular barriers. In addition, it can protect siRNA from degradation by RNaseA and promote the cellular uptake and endosomal escape of si*TGF-β1*. Stimulation by BLM can lead to the expression of key factors involved in EMT in cellular or animal models. Meanwhile, si*TGFβ1*-LNPs can delay the activation of fibroblasts and cause the downregulation of *CTGF* factor by downregulating *TGF-β1*, thereby delaying the production of *Fn* and *Collagen*, delaying tissue stiffness and structural abnormalities. It can also cause the downregulation of inflammation-related factors such as *IL-6* and *IL-17* as well as *α-SMA*, delaying the transformation of fibroblasts into myofibroblasts. Ultimately, it delays the progression of pulmonary fibrosis. si*TGFβ1*-LNPs can effectively deliver target siRNA to lungs, resulting in selective gene silencing, which has been demonstrated to remarkably ameliorate the deterioration in the murine models of PF. This study provides a new approach for treating PF by targeting *TGF-β1* silencing. Moreover, in this study, the administration method is through nebulization, which can increase the drug concentration in the lungs and reduce systemic side effects.

## Figures and Tables

**Table 1:** The list of si*Fluc*-LNPs formulations.

Batch	Model	MC3 (%) *^1^	DSPC (%) *^1^	DMG-PEG2000 (%) *^1^	N/P *^2^	Cholesterol (%) *^1^
1	0	50	10	3	3.25	37
2	−00+	40	10	3	5	47
3	+00−	60	10	3	1.5	27
4	−0+0	40	10	5	3.25	45
5	+0−0	60	10	1	3.25	29
6	0−0−	50	7	3	1.5	40
7	00+−	50	10	5	1.5	35
8	0−+0	50	7	5	3.25	38
9	0+−0	50	13	1	3.25	36
10	+00+	60	10	3	5	27
11	+−00	60	7	3	3.25	30
12	0++0	50	13	5	3.25	32
13	00−−	50	10	1	1.5	39
14	0+0+	50	13	3	5	34
15	00−+	50	10	1	5	39
16	0−−0	50	7	1	3.25	42
17	0	50	10	3	3.25	37
18	0+0−	50	13	3	1.5	34
19	00++	50	10	5	5	35
20	+0+0	60	10	5	3.25	25
21	−+00	40	13	3	3.25	44
22	0−0+	50	7	3	5	40
23	00−0	50	10	1	3.25	39

*^1^: The numbers in the table represent the relative content of each LNP component in the entire formulation. *^2^: The numbers in the “N/P” column represent the molar ratio of nitrogen (N) to phosphorus (P) in the formulation.

**Table 2:** Primer sequences used for qRT–PCR.

Gene Name	Primer (5′-3′)
*M-IL-6-F*	TACCACTTCACAAGTCGGAGGC
*M-IL-6-R*	CTGCAAGTGCATCATCGTTGTTC
*M-IL-17A-F*	CAGACTACCTCAACCGTTCCAC
*M-IL-17A-R*	TCCAGCTTTCCCTCCGCATTGA
*M-Fn-F*	CCCTATCTCTGATACCGTTGTCC
*M-Fn-R*	TGCCGCAACTACTGTGATTCGG
*mα-SMA-F*	GTACCCAGGCATTGCTGACA
*mα-SMA-R*	GAGGCGCTGATCCACAAAAC
*mCollagen I-F*	GTCAGACCTGTGTGTTCCCTACTCA
*mCollagen I-R*	TCTCTCCAAACCAGACGTGCTTC
*M-CTGF-F*	TCCGGACACCTAAAATCGCC
*M-CTGF-R*	TTCATGATCTCGCCATCGGG
*M-TGFβ1-F*	TACCTGAACCCGTGTTGCTCTC
*M-TGFβ1-R*	GTTGCTGAGGTATCGCCAGGAA
*M-GAPDH-F*	CTTTGTCAAGCTCATTTCCTGG
*M-GAPDH-R*	TCTTGCTCAGTGTCCTTGC

## Data Availability

The data presented in this study are available within the article.

## Supplementary Materials

The following supporting information can be downloaded at https://www.mdpi.com/article/10.3390/pharmaceutics17020157/s1, Figure S1: The lipid structures.
