# Supplementary figures and images for: Engineering Lipid Nanoparticles to Enhance Intracellular Delivery of Transforming Growth Factor-Beta siRNA (siTGF-β1) via Inhalation for Improving Pulmonary Fibrosis Post-Bleomycin Challenge
Source: Pharmaceutics. 2025 Jan 24;17(2):157. doi: 10.3390/pharmaceutics17020157 (PMC11859093; doi:10.3390/pharmaceutics17020157)

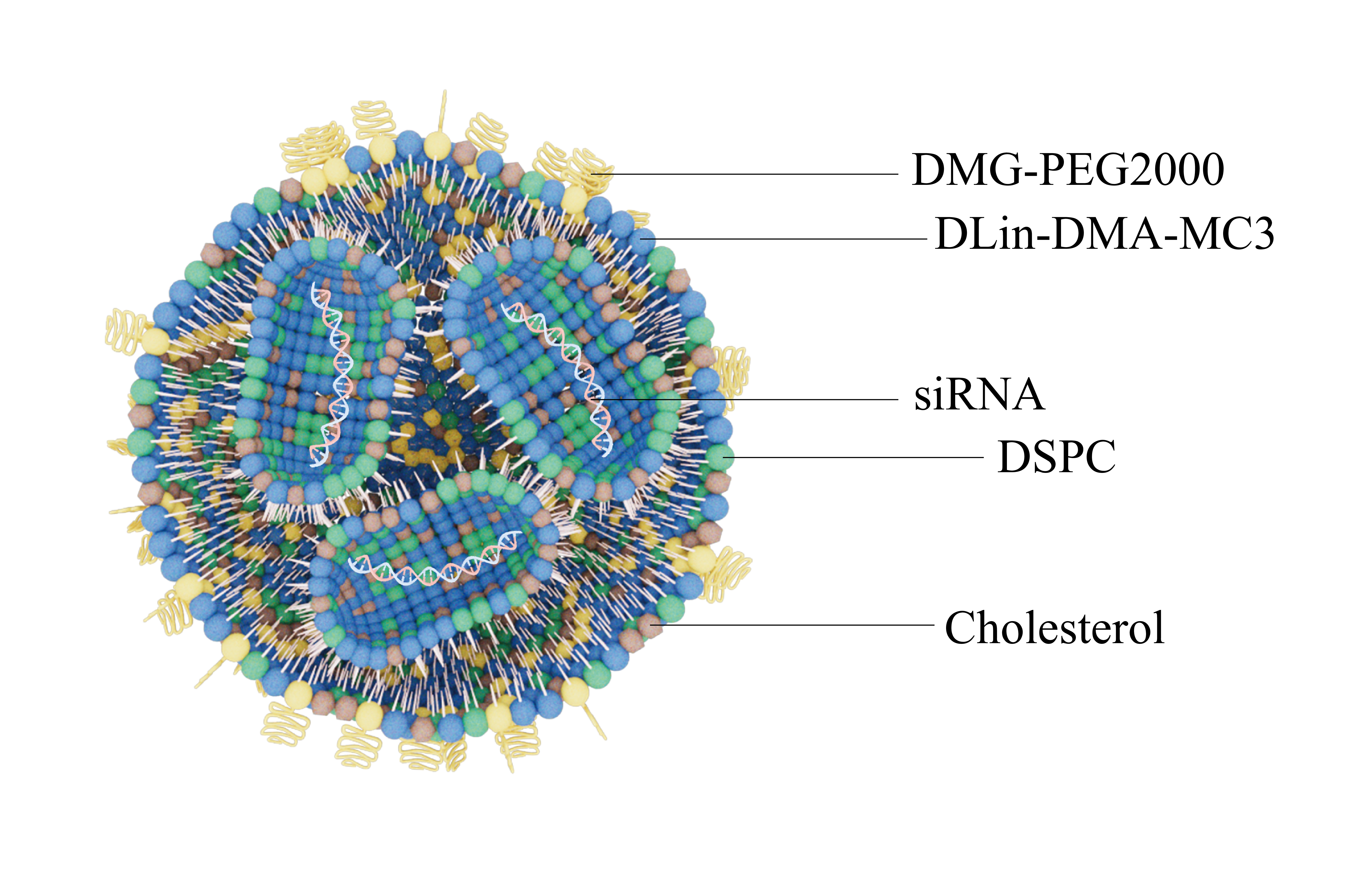

Supplement: Supplementary file 1 [file pharmaceutics-17-00157-s001.zip › pharmaceutics-3423289-supplementary.tiff]
